# Essential Role of the Small GTPase Ran in Postnatal Pancreatic Islet Development

**DOI:** 10.1371/journal.pone.0027879

**Published:** 2011-11-17

**Authors:** Fang Xia, Takehiko Dohi, Nina M. Martin, Christopher M. Raskett, Qin Liu, Dario C. Altieri

**Affiliations:** 1 Prostate Cancer Discovery and Development Program, The Wistar Institute, Philadelphia, Pennsylvania, United States of America; 2 Department of Cancer Biology, University of Massachusetts Medical School, Worcester, Massachusetts, United States of America; 3 Center for Computational and Systems Biology, The Wistar Institute, Philadelphia, Pennsylvania, United States of America; Universita Magna-Graecia di Catanzaro, Italy

## Abstract

The small GTPase Ran orchestrates pleiotropic cellular responses of nucleo-cytoplasmic shuttling, mitosis and subcellular trafficking, but whether deregulation of these pathways contributes to disease pathogenesis has remained elusive. Here, we generated transgenic mice expressing wild type (WT) Ran, loss-of-function Ran T24N mutant or constitutively active Ran G19V mutant in pancreatic islet β cells under the control of the rat insulin promoter. Embryonic pancreas and islet development, including emergence of insulin^+^ β cells, was indistinguishable in control or transgenic mice. However, by one month after birth, transgenic mice expressing any of the three Ran variants exhibited overt diabetes, with hyperglycemia, reduced insulin production, and nearly complete loss of islet number and islet mass, in vivo. Deregulated Ran signaling in transgenic mice, adenoviral over-expression of WT or mutant Ran in isolated islets, or short hairpin RNA (shRNA) silencing of endogenous Ran in model insulinoma INS-1 cells, all resulted in decreased expression of the pancreatic and duodenal homeobox transcription factor, PDX-1, and reduced β cell proliferation, in vivo. These data demonstrate that a finely-tuned balance of Ran GTPase signaling is essential for postnatal pancreatic islet development and glucose homeostasis, in vivo.

## Introduction

As a member of the Ras family of small GTPases [1], the Ran protein orchestrates a multitude of cellular responses, including nucleo-cytoplasmic shuttling [2], various aspects of mitosis [3], and other cytoplasmic transport mechanisms in specialized cell types [4]. These functions require regulated subcellular compartmentalization of Ran [3], spatial control of its guanine nucleotide cycling [5], and a finely-tuned balance involving plethora of Ran regulatory molecules that monitor the guanine-nucleotide state [6].

Ran signaling is highly evolutionary conserved, and is thought to be essential for cellular homeostasis [6]. However, except for transformed cells, where Ran is frequently over-expressed [7], controls the distribution [8], and/or stability [9], [10] of various *cancer* genes, and correlates with unfavorable outcome [11], [12], a mechanistic link between deregulated Ran signaling and disease pathogenesis has not been determined.

In this study, we generated transgenic mice that express wild type (WT) Ran, the Ran loss-of-function mutant T24N, or the Ran gain-of-function mutant G19V [6] in insulin-producing pancreatic islet β cells. Unexpectedly, we found that deregulated Ran signaling under these conditions dramatically impairs postnatal, but not embryonic islet development, triggering hypoinsulinemia, reduced β cell proliferation and overt diabetes, in vivo.

## Materials and Methods

### Plasmid construction and generation of transgenic mice

All experiments involving animals were approved by an Institutional Animal Care and Use Committee. A full-length human Ran WT cDNA or cDNA encoding the Ran mutant T24N or G19V was fused to an HA tag at the 5′ end, and cloned into *BamHI* and *SpeI* sites downstream of the Rat Insulin Promoter (RIP) [13] in pBluescript II KS, containing SV40 polyadenylation sequences at the 3′ end. Each RIP-HA-Ran construct (WT, T24N or G19V) was confirmed by DNA sequencing, purified by ion exchange chromatography (Qiagen, Valencia, CA), and microinjected (5 ng/ml) into C57Bl/6 embryos that were implanted into syngeneic recipient pseudopregnant females, as described [14]. Littermates were screened by PCR of tail genomic DNA using primers (10 pmol) corresponding to RIP-HA (forward, 5′-CTCGAGGGCTGCAGGAATTCGATA-3′; reverse 5′-GCCTTCACTTTCCTGTCCTTAATA-3′) or RIP-Ran (forward 5′-TGGACTA TAAAGCTAGTGGGGATT-3′; reverse, (5′-GCTGTGTCCCATACATTGAACTTA-3′) sequences. PCR reactions (35 cycles) were carried out at 95°C for 1 min, 56°C for 1 min and 72°C for 1 min plus a 10 min extension at 72°C. Colonies from independent transgene-positive founder mice or control littermates were established, and bred with C57Bl/6 mice. Two independent colonies per each condition were analyzed for blood glucose levels with comparable results. Plasmid adenoviral (pAd) constructs encoding GFP-Ran-WT, GFP-Ran-T24N or GFP-Ran-G19V were generated using the pAdEasy system, as described previously [14].

### Cell culture, antibodies, and Western blotting

The rat insulinoma cell line INS-1 was the kind gift of R.S. Sherwin (Yale University School of Medicine, New Haven, CT), and was maintained in culture as described [15]. The following antibodies to Ran (Novus Biologicals, Cell Signaling, Santa Cruz), HA (Santa Cruz, Roche Applied Science), insulin (Invitrogen), glucagon (Dako), somatostatin (Dako), Ki-67 (Dako), PDX-1 (Upstate Biotechnology), or β-actin (Sigma-Aldrich), were used. Restriction enzymes were purchased from New England BioLabs. Pancreas or liver tissues isolated from non-transgenic (non-TG) or Ran transgenic mice were washed in PBS (pH 7.2), suspended in 4 to 5 volumes of cold lysis buffer containing 50 mM Tris-HCl (pH 7.5), 150 mM NaCl, 5 mM EDTA, 50 mM NaF, 0.5% Nonidet P-40, plus protease inhibitors (Roche Applied Science). After 30-min incubation at 4°C, tissue extracts were cleared by centrifugation at 14,000 rpm for 20 min at 4°C, and analyzed by Western blotting. Alternatively, INS-1 cells were transduced with a lentivirus expressing control pLKO or Ran-directed short hairpin RNA (shRNA) (Open Biosystems), selected with 1 µg/ml puromycin, and analyzed by Western blotting. Protein content was determined using a BCA-200 protein assay kit (Pierce).

### Pancreatic islet isolation

Pancreatic islets were harvested from control or Ran transgenic mice by collagenase P (1 mg/ml) (Sigma-Aldrich) perfusion, as described [14]. After filtration through a 100 µm cell strainer, islets were hand-picked under a dissecting microscope. Islets isolated from non-TG mice were transduced in vitro with pAd-GFP or pAd-GFP-Ran-WT, pAd-GFP-Ran-G19V or pAd-GFP-Ran-T24N, as described previously [14], and analyzed after 48 h for GFP expression by fluorescence microscopy or endogenous PDX-1 levels, by Western blotting.

### Islet glucose stimulation, in vitro

Islets isolated from non-TG or transgenic mice expressing Ran-WT, Ran-G19V or Ran-T24N (7 mice/group) at 2–5 mo of age were plated onto six-well plates (20 islets/well), and cultured in 2 ml DMEM medium for 24 h. Cells were suspended in 1 ml OPTI-MEM for 4 h, separately incubated with 5 mM or 16.7 mM D-glucose, and 200 µl aliquots of the supernatants were collected after 0.5, 1, and 2 h for determination of insulin levels, as described [14]. Similar experiments were carried out with parental or shRNA-transduced stable INS-1 clones.

### Glucose and insulin secretion

Non-TG or Ran transgenic mice were maintained with feeding *ad libitum*, or, alternatively, under fasting conditions for 16 h. At the end of the fasting period, thirty µl of blood was collected from the tail vein, and analyzed for glucose or insulin content using an ELISA assay kit (Crystal Chem).

### Immunohistochemistry

Pancreas tissues from non-TG or Ran transgenic mice were fixed in 10% neutral-buffered formalin, dehydrated through graded ethanol passages, and embedded in paraffin wax. Five µm-thick tissue sections were cut, put on high-adhesive slides, and stained with hematoxylin and eosin (H&E). Immunohistochemical staining was performed as described [14], using primary antibodies to insulin (1∶500) or PDX-1 (1∶200). Quantification of tissue staining was carried out by morphometry, as described [14]. For islet cell proliferation or apoptosis, pancreas sections were incubated with an antibody to Ki-67 (1∶100), or analysed for terminal deoxynucleotidyl transferase-mediated dUTP nick end labeling (TUNEL, Roche), respectively, as described [14], and positively stained islet cells were quantified. Images were collected on an Olympus microscope with on-line charge-coupled device camera. Islet surface area normalized to mm^2^ or islet number was quantified from insulin-labeled, serial pancreas sections by morphometry, as described [14].

### Statistical analysis

Data were analyzed by two-sided unpaired t-tests using a GraphPad software package (Prism 4.0) for Windows. In some experiments, ANOVA and post-hoc multiple tests with Bonferroni procedure or Mann-Whitney tests were used for data analysis. A *p* value of 0.05 was considered as statistically significant.

## Results

### Characterization of Ran-β cell transgenic mice

**Figure 1:**
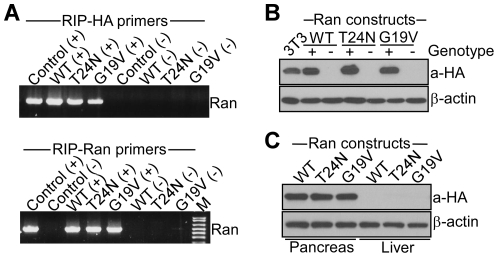
Characterization of Ran transgenic mice. *A*, Tail DNA was extracted from transgenic mouse lines expressing Ran-WT, Ran T24N or Ran G19V in pancreatic β cells under RIP control, and PCR products were amplified using RIP-HA (*top*) or RIP-Ran (*bottom*) primers. (+) or (−) refers to Ran-positive or –negative genotype, or positive or negative control primers for the amplification reaction. M, molecular weight markers. *B*, Pancreas tissues extracted from the various mouse cohorts genotyped for the presence (+) or absence (−) of Ran transgenes were analyzed by Western blotting. 3T3, extracts from NIH3T3 cells transiently transfected with HA-Ran cDNA used as control. a-HA, antibody to HA. *C*: Pancreas or liver tissues were isolated from PCR-confirmed Ran transgenic mice, and analyzed by Western blotting. β-actin was used as a loading control.

We generated transgenic mice that express HA-tagged Ran WT, a Ran T24N mutant that is unable to hydrolyze GTP, or a Ran G19V mutant with constitutively active GTPase function [6] in insulin-producing pancreatic β cells [13]. PCR products corresponding to Ran-WT, Ran-T24N or Ran-G19V construct were amplified from tail DNA using transgene-specific primers for RIP-HA (Fig. 1*A*, *top*), or RIP-Ran (Fig. 1A, *bottom*) sequences. Pancreas extracts from PCR-confirmed transgenic mice reacted with an antibody to HA, whereas PCR-negative samples were unreactive (Fig. 1B). In addition, HA-reactive material was demonstrated in pancreas, but not liver extracts of PCR-confirmed Ran transgenic mice (Fig. 1C), confirming tissue-specific expression of the various cDNA constructs. Endogenous Ran levels in pancreas of control littermates were negligible, by Western blotting of isolated islet tissues (see below).

### Deregulated Ran signaling in β cells impairs glucose metabolism and islet maintenance

**Figure 2:**
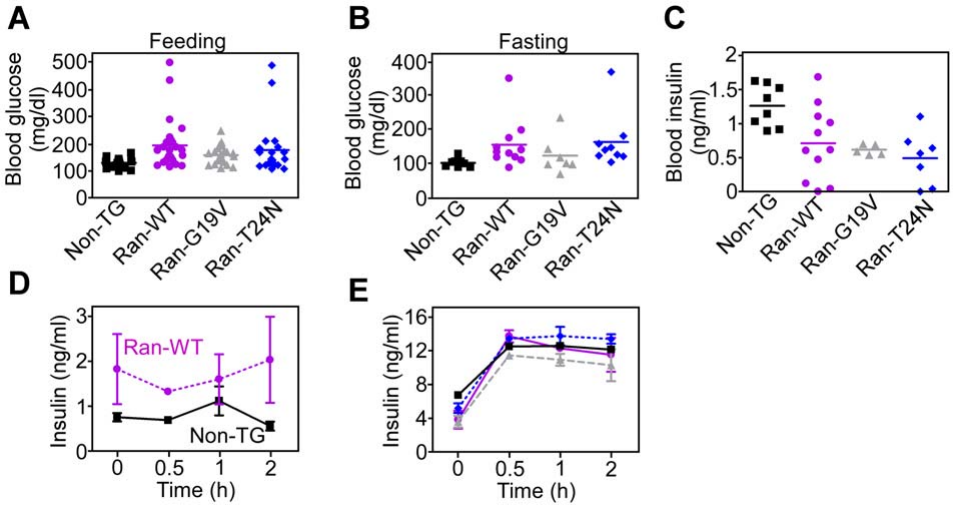
Transgenic expression of Ran impairs glucose metabolism. *A and B*, The indicated PCR-confirmed transgenic mice were analyzed for blood glucose content at 2 mo of age under ad libitum feeding (*A*) or fasting (*B*) conditions. Non-TG, non-transgenic mice. Glucose concentrations (mg/dl) in each group in *A* (number of mice in parentheses), and statistical analyses (unpaired *t* test) are as follows: Non-TG (n = 24), 125±2; Ran-WT (n = 26), 189.4±17.8, p = 0.013; Ran-G19V (n = 24), 153.7±6.5, p = 0.003; Ran-T24N (n = 21), 172.4±21.1, p = 0.02. Statistical data re-analysis of the groups in *A* using ANOVA and post-hoc multiple tests with Bonferroni procedure was as follows: Ran-WT, p<0.0001; Ran-G19V, 0.017; Ran-T24N, p = 0.029. *C*, The indicated non-TG or Ran transgenic mice were analyzed for blood insulin concentrations. Insulin levels (ng/ml) in each group (number of mice in parentheses), and statistical analyses (unpaired *t* test) are as follows: Non-TG (n = 8), 1.26±0.1; Ran-WT (n = 11), 0.7±0.16, p = 0.019; Ran-G19V (n = 5), 0.61±0.03, p = 0.0008; Ran-T24N (n = 7), 0.49±0.14, p = 0.0009. Statistical data re-analysis using ANOVA and post-hoc multiple tests with Bonferroni procedure was as follows: Ran-WT, p = 0.023; Ran-G19V, p = 0.031; Ran-T24N, p = 0.004. One outlier mouse in the Ran-G19V group with aberrantly high insulin level (2.35 ng/ml) was excluded from the analysis. For panels *A–C*, each point corresponds to an individual mouse. *D*, Islets (20/well) isolated from non-TG or Ran-WT transgenic mice were incubated with 5 mM D-glucose, and analyzed for insulin release in the supernatant at the indicated time intervals. Mean±SD of replicates. *E*, Islets (20/well) isolated from non-TG (*black*) or Ran-WT (*purple*), Ran-G19V (*grey*) or Ran-T24N (*blue*) transgenic mice were incubated with 16.7 mM glucose, and analyzed for insulin release in the supernatant at the indicated time intervals. Mean±SD of replicates.

**Figure 3:**
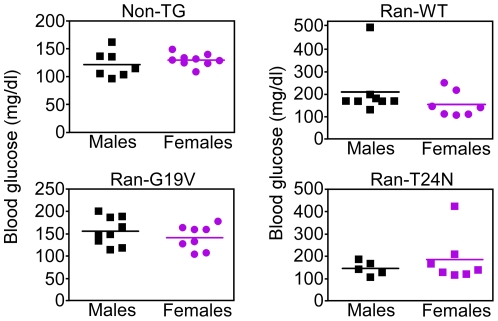
Gender analysis of the diabetic phenotype in Ran transgenic mice. The indicated non-TG or Ran transgenic mice were analyzed for gender differences in blood glucose levels at 2 mo of age. Each point corresponds to an individual mouse.

Ran transgenic mice did not present overt developmental defects, were born at expected rates and were fertile. However, by 1–2 mo of age, subsets of transgenic mice expressing Ran-WT (57%), Ran-G19V (58%) or Ran-T24N (42%) exhibited blood glucose levels >150 mg/dl under *ad-libitum* feeding (Fig. 2A), or fasting (Fig. 2B) conditions. In contrast, non-transgenic mice had blood glucose levels of 125±.3.2 mg/dl, which was considered within the normal range. Hyperglycemia in Ran transgenic mice was associated with reduced blood insulin levels, compared to non-transgenic littermates (Fig. 2C). In addition, pancreatic islets isolated from non-transgenic mice responded to glucose stimulation with a transient increase in insulin release, peaking at 1 h, and returning to baseline 2 h after challenge (Fig. 2D). In contrast, islets from representative asymptomatic Ran-WT transgenic mice (Fig. 1C) had constitutively higher basal insulin levels, potentially reflecting a compensatory response to decreasing postnatal β cell mass (see below), which were not further modulated by stimulation with low glucose concentrations (5 mM) (Fig. 2D). Conversely, islets isolated from all three transgenic mouse lines expressing Ran-WT, Ran-G19V or Ran-T24 responded to stimulation with higher glucose concentrations (16.7 nM) with an increase in insulin release that peaked 1 h after challenge (Fig. 2E). Most diabetic Ran transgenic mice died of hyperglycemia by 4–6 mo of age and none was alive by 8 mo. Different from other mouse models of diabetes [16], [17], no gender-specific differences were observed in the development of hyperglycemia in Ran transgenic mice (Fig. 3).

**Figure 4:**
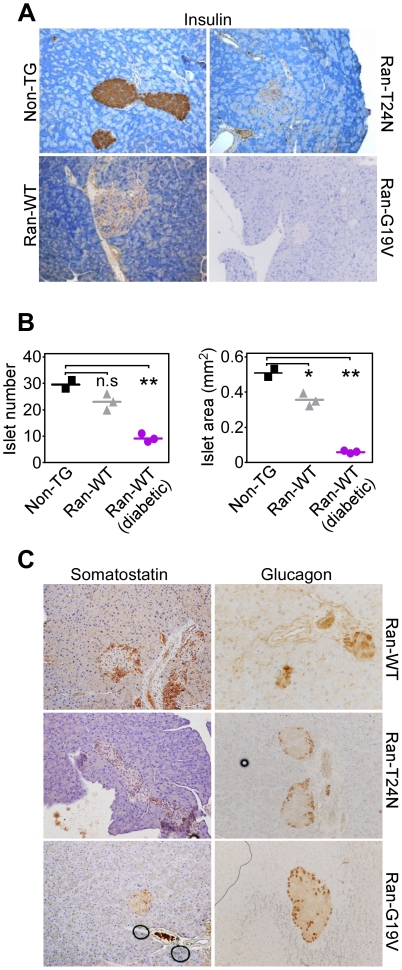
Defective islet development in Ran transgenic mice. *A*, Pancreas tissues from non-TG, Ran-WT, Ran-G19V or Ran-T24N transgenic mice were analyzed by H&E or immunohistochemical staining with an antibody to insulin. *B*, Pancreas sections from non-TG, asymptomatic Ran-WT or diabetic Ran-WT transgenic mice were analyzed for islet number or islet surface area by morphometry of insulin-stained areas. Islet number, non-TG (n = 2), 29.5±1.5; Ran-WT (n = 3), 23±1.73, n.s., not significant; Ran-WT diabetic (n = 3), 9.33±0.88, **, p = 0.001; islet surface area, non-TG (n = 2), 0.51±0.02; Ran-WT (n = 3), 0.35±0.021, *, p = 0.017; Ran-WT diabetic (n = 3), 0.057±0.004, **, p<0.0001. *C*, Pancreas tissues from Ran-WT, Ran-G19V or Ran-T24N transgenic mice were analyzed by immunohistochemical staining with antibodies to somatostatin or glucagon.

Pancreas tissue isolated from non-TG mice contained morphologically developed endocrine islets that stained intensely positive for insulin (Fig. 4A). In contrast, by 2 mo of age, symptomatic transgenic mice expressing any of the three Ran variants, WT, G19V or T24N in pancreatic β cells exhibited largely undetectable insulin staining, in situ (Fig. 4A). Consistent with these data, overtly diabetic Ran-WT transgenic mice at 2 mo of age exhibited nearly complete loss of pancreatic islet number and islet mass, compared to control littermates (Fig. 4B). In contrast, asymptomatic Ran-WT transgenic mice (Fig. 2A) of comparable age had intermediate defects in postnatal islet development, with partial reduction in islet number and islet mass, compared to the control group (Fig. 4B). Conversely, production of glucagon by pancreatic α cells, or somatostatin by δ cells was unaffected in all three transgenic mouse lines expressing Ran-WT, Ran-G19V or Ran-T24N (Fig. 4C).

### Postnatal defect of pancreatic islet maintenance in Ran transgenic mice

**Figure 5:**
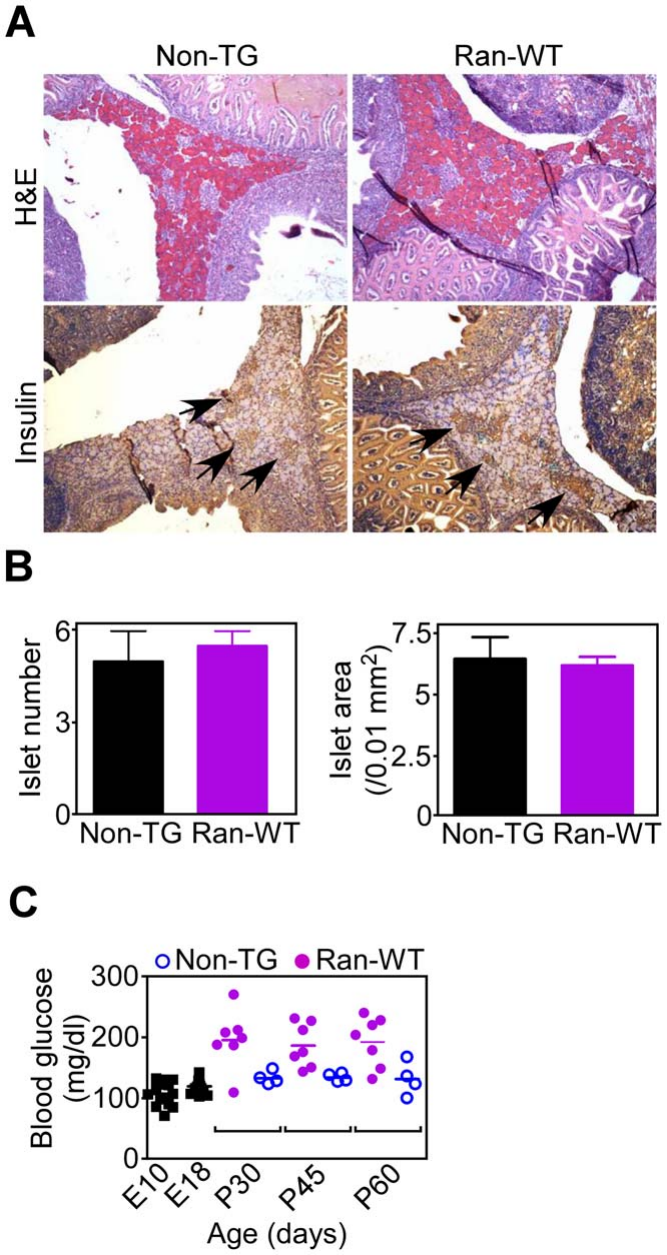
Postnatal defect in islet development in Ran transgenic mice. *A*, E18 non-TG or Ran-WT embryos were analyzed by H&E or immunohistochemical staining with an antibody to insulin. *Arrows*, insulin-stained islets. *B*, Islet number (*left*), or islet surface area (*right*), was quantified in E18 non-TG or Ran-WT transgenic embryos by morphometry of insulin-stained sections. The differences among groups are not statistically significant. *C*, Littermates of Ran-WT diabetic mice crossed with normal mice were analyzed for changes in blood glucose levels at the indicated time intervals. E, embryonic; P, postnatal. Glucose levels in PCR-confirmed non-TG (*open circles*) or Ran-WT transgenic (*closed circles*) mice are shown. Each point corresponds to an individual mouse. Glucose concentrations (mg/dl) in each group (number of mice in parentheses), and statistical analyses *versus* E18 values are as follows: E10 (n = 15), 108.7±4.93; E18 (n = 15), 120.7±2.7; P30 Ran-WT (n = 7), 196.3±17.8, p<0.0001; P30 non-TG (n = 4), 134±5.4; P45 Ran-WT (n = 7), 187.1±13.5, p<0.0001; P45 non-TG (n = 4), 134.8±3.4; P60 Ran-WT (n = 7), 193.1±15.5, p<0.0001; P60 non-TG (n = 4), 132.5±13.9. Statistical data re-analysis using Mann-Whitney test was as follows: P30 Ran-WT, p = 0.013; P45 Ran-WT, p = 0.0006; P60 Ran-WT, p = 0.0009.

PCR-confirmed non-TG E18 embryos contained morphologically discrete endocrine islets, which stained positive for insulin, by immunohistochemistry (Fig. 5A). Ran-WT E18 embryos also revealed the presence of insulin-stained islets (Fig. 5A), quantitatively indistinguishable for number and surface area from those of non-TG embryos (Fig. 5B). Metabolically, embryos collected at E10 or E18 had normal blood glucose levels (Fig. 5C). However, by postnatal d 30 (P30), a subset of mice (63%) developed hyperglycemia, which was maintained at analyses on P45 and P60 (Fig. 5C). Six out of seven of these diabetic mice were confirmed positive for the Ran-WT transgene by PCR, whereas none of the non-transgenic mice showed hyperglycemia (Fig. 5C).

**Figure 6:**
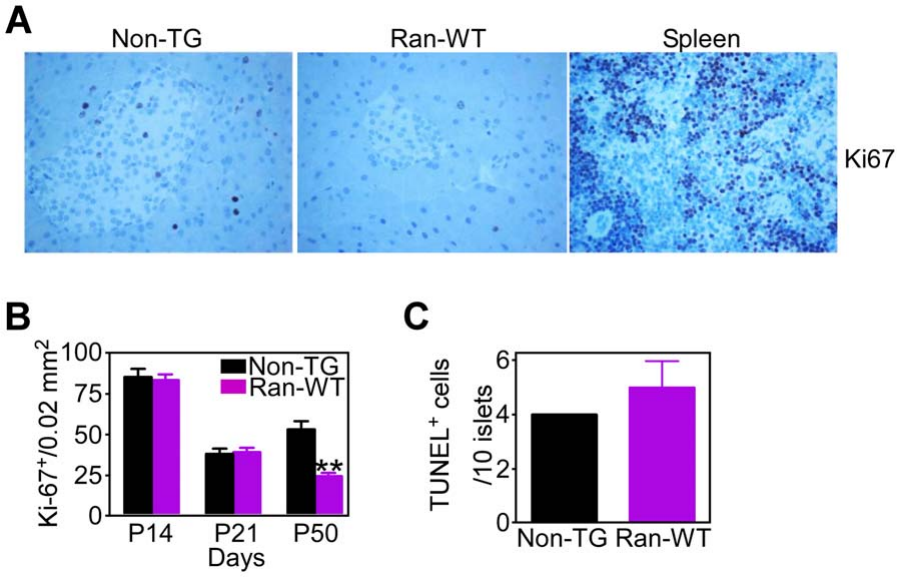
Defective islet cell proliferation in Ran transgenic mice. *A*, Pancreas section from non-TG or Ran-WT transgenic mice were harvested at the indicated postnatal (P) age and analyzed for Ki-67 reactivity, by immunohistochemistry. Sections from normal mouse spleen were used as control. *B*, The number of Ki-67^+^ cells/islet surface area (0.02 mm^2^) was quantified by morphometry. **, p = 0.0081. *C*, Pancreas section from non-TG or Ran-WT transgenic mice (P50) were analyzed for TUNEL reactivity and the numbers of positive cells was quantified. Mean±SD.

Time-course analysis of islet cell proliferation revealed no significant differences in the number of Ki-67^+^ islet cells between transgenic and non-transgenic specimens at P14 or P21 (Fig. 6A, B). In contrast, islet cell proliferation was significantly reduced in Ran-WT transgenic mice by P50 (Fig. 6A, B). Conversely, no differences in islet cell apoptosis were observed between transgenic and non-transgenic mice, by TUNEL staining of pancreas sections at comparable age (Fig. 6C).

### Deregulated Ran signaling affects PDX-1 expression

**Figure 7:**
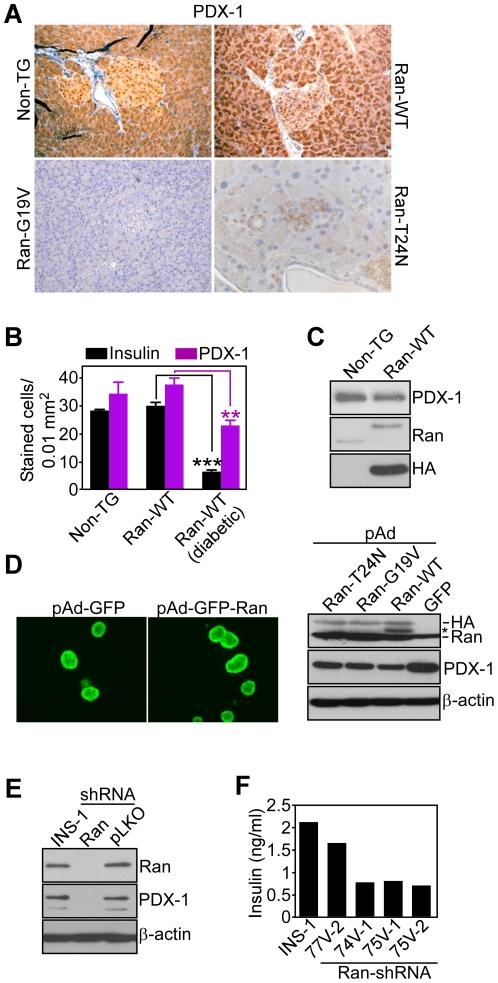
Deregulated PDX-1 expression in Ran-targeted cells. *A*, Pancreas sections from non-TG, Ran-WT, Ran-G19V or Ran-T24N transgenic mice were stained with an antibody to insulin or PDX-1, by immunohistochemistry. *B*, The number of insulin- or PDX-1-stained cells was quantified by morphometry in the indicated surface area. Insulin^+^ cells, non-TG (n = 2), 28.2±0.4; Ran-WT (asymptomatic, n = 3), 29.8±1.2; Ran-WT (diabetic, n = 3), 6.2±0.6, ***, p<0.0001; PDX-1^+^ cells, Non-TG (n = 2), 34.2±4.1; Ran-WT (asymptomatic, n = 3), 37.5±2.3; Ran-WT (diabetic, n = 3), 22.8±1.8, **, p = 0.007. *C*, Islets from 2 mo-old non-TG or a Ran-WT transgenic mouse were analyzed by Western blotting. *D*, Pancreatic islets isolated from non-TG mice were transduced ex vivo with control pAd-GFP or pAd-GFP-Ran-WT, pAd-GFP-Ran-G19V or pAd-GFP-T24N and analyzed after 48 h by fluorescence microscopy for GFP expression (*left*), or Western blotting (*right*). *, non-specific. *E*, INS-1 cells were left untreated (INS-1) or transduced with control lentivirus (pLKO) or lentivirus encoding Ran-directed shRNA (Ran, 74V1), and analyzed by Western blotting. *F*, Parental INS-1 cells or four independent clones of INS-1 cells stably transduced with Ran-directed shRNA (77V-2, 74V-1, 75V-1, 75V-2) were analyzed for changes in insulin release in the supernatant. Representative experiment out of at least two independent determinations.

In addition to the loss of insulin staining (Fig. 4A), pancreas tissue from overtly diabetic transgenic mice expressing Ran-WT, Ran-G19V or Ran-T24N at 2 mo of age exhibited significantly decreased expression of the pancreatic and duodenal homeobox transcription factor, PDX-1 [18], compared to non-transgenic mice or asymptomatic Ran-WT transgenic animals, by quantitative morphometry of immunohistochemically-stained areas (Fig. 7A, B). Similar results were obtained by Western blotting analysis of PDX-1 expression in pancreas tissues isolated from diabetic Ran-WT transgenic mice, compared to control, non-TG littermates (Fig. 7C). We next used two independent approaches to examine the dependence of PDX-1 expression on Ran signaling. First, we transduced pancreatic islets isolated from non-TG mice with GFP-encoding adenoviral constructs (pAd) expressing HA-tagged Ran-WT, Ran-G19V or Ran-T24N. In these experiments, adenoviral transduction of isolated islets was associated with GFP reactivity, by fluorescence microscopy (Fig. 7D, *left*), and expression of HA-tagged recombinant Ran proteins, by Western blotting (Fig. 7D, *right*). In addition, adenoviral over-expression of all three Ran isoforms in isolated islets resulted in reduced levels of endogenous PDX-1, compared to pAd-GFP transduction, by Western blotting (Fig. 7D, *right*), thus mirroring the phenotype of Ran transgenic mice (Fig. 7B, C). Second, we targeted Ran by gene silencing approaches in model insulinoma INS-1 cells. Due to their transformed phenotype, these cells express elevated levels of endogenous Ran, in agreement with previous observations [9]. In these experiments, INS-1 cells stably transfected with lentivirus expressing Ran-directed shRNA exhibited nearly complete loss of endogenous Ran levels, which was associated with loss of PDX-1 expression, by Western blotting (Fig. 7E). In control experiments, INS-1 clones stably transduced with a control non-targeting lentivirus (pLKO) had endogenous levels of Ran or PDX-1 comparable to those of parental cells (Fig. 7E). In addition, stable shRNA silencing of Ran in four independent INS-1 clones was associated with reduced insulin production, compared to parental INS-1 cells (Fig. 7F).

## Discussion

In this study, we have shown that transgenic expression of functional variants of the Ran GTPase [6], [19] in pancreatic β cells induces catastrophic defects of postnatal islet development, resulting in hypoinsulinemia, and an overt diabetic phenotype. Although a subset of Ran transgenic mice were asymptomatic (∼40%) at the time of diabetic onset (2 mo), these animals also revealed reduced islet number and islet mass, compensatory hyperinsulinemia, and impaired insulin response to glucose stimulation of isolated islets, in vitro. Mechanistically, deregulated Ran signaling in islet β cells was associated with decreased expression of the transcriptional regulator of islet development, PDX-1 [18], and impaired postnatal islet cell proliferation, but not apoptosis, in vivo.

GTP-binding proteins, or G proteins, function as important effectors of glucose homeostasis [20], orchestrating cytoskeletal remodeling [21], and vesicle fusion [22] for insulin secretion, as well as docking of insulin secretory granules at the plasma membrane [23], [24]. Genetic models support this view, as deletion of the Rab3A [25], or Rab27a [26] G protein in mice results in hyperglycemia, hypoinsulinemia and glucose intolerance, whereas transgenic expression of a dominant negative Rac1 mutant in β cells impairs islet morphogenesis, potentially by interfering with migration of pancreatic β cells away from the ductal epithelium [27].

Conversely, a role of the Ran G protein [6], [19] in glucose homeostasis has not been previously described. Here, transgenic expression of three functionally different Ran variants in β cells caused a comparable phenotype of overt diabetes, suggesting that a finely-tuned balance of Ran GTPase signaling [6], [19] is required for β cell maintenance, in vivo. Mechanistically, this pathway was associated with decreased expression of the transcriptional regulator of islet development, PDX-1 [18]. A large body of literature points to an essential role of PDX-1 in pancreas and islet formation in mice [28], [29], and humans [30], orchestrating the differentiation and expansion of precursor cells within the pancreatic buds (E8.5–9.5), formation of acinar cells and islets (E12.5), and completion of secondary transition (E14.5–E15.5) [17], [31]. There is also evidence that persistent PDX-1 activity is required to maintain a functional β cell mass in the adult pancreas [32], [33], where its expression becomes restricted to β, but not acinar cells [34]. Accordingly, post-developmental haploinsufficiency of PDX-1 causes an overtly diabetic phenotype in mice [35], [36], mechanistically associated with increased β cell apoptosis [35], [36], loss of downstream PDX-1 target genes implicated in glucose-stimulated insulin transcription [37], [38], and impaired β cell proliferation/regeneration [39]. Together, these data are consistent with a model in which loss of PDX-1 expression in Ran transgenic mice triggers developmental defects of postnatal islet formation, associated with decreased islet cell proliferation (i.e. β cell neogenesis), in situ [40]. Conversely, deregulated Ran signaling did not affect islet formation in E10 or E18 transgenic embryos, suggesting that PDX-1 activity at this developmental stages [31] does not depend on Ran GTPase function.

Additional work is required to conclusively elucidate how Ran GTPase activity controls PDX-1 expression and/or function in mature β cells [32], [33]. This pathway may not involve modulation of PDX-1 nucleo-cytoplasmic shuttling [41], as shRNA silencing of Ran in INS-1 cells comparably depleted PDX-1 in cytosolic and nuclear compartments, by Western blotting of isolated subcellular fractions (our unpublished observations). Instead, data presented here suggest that Ran may help maintaining PDX-1 protein stability in postnatal β cells, similar to its function on other downstream Ran target proteins, including survivin [9], or TPX-2/Aurora A [10]. It is also plausible that other Ran target proteins contribute to postnatal islet development, a possibility suggested by the partial decrease in PDX-1 expression in Ran transgenic mice, as opposed to the nearly complete loss of insulin production under these conditions, in vivo.

In summary, we have identified the small GTPase, Ran as an essential effector of postnatal islet development. The requirements of this pathway differ from previously known examples of G protein regulation of β cell morphogenesis [27], or insulin secretion [20], and involve a temporal control of postnatal PDX-1 expression [18], and mature β cell proliferation. Future studies will elucidate the functional partners of Ran GTPase signaling in postnatal islet development, and PDX-1-dependent gene expression.
